# Psychometric comparison of five short forms of the DASS in Mexican university students: implications for emotional distress screening

**DOI:** 10.3389/fpsyg.2026.1808531

**Published:** 2026-05-01

**Authors:** Luis Hernando Silva Castillo, Enrique Hernández-Rosas

**Affiliations:** 1 Departamento de Psicología Educación y Salud, Western Institute of Technology and Higher Education, Tlaquepaque, Mexico; 2 Posgrado en Ciencias del Comportamiento, Universidad de Guadalajara, Guadalajara, Mexico

**Keywords:** anxiety, depression, Depression Anxiety Stress Scales, mass screening, psychological, psychometrics, stress

## Abstract

**Background:**

Early identification of emotional distress in university students requires brief yet psychometrically sound instruments that enable efficient screening without sacrificing interpretive precision. Although the Depression, Anxiety and Stress Scales-21 (DASS-21) is widely used, its length has prompted the development of several short forms. However, direct comparative evidence to guide the selection of these versions in applied settings, particularly in Mexican populations, remains limited.

**Methods:**

This study compared five versions of the DASS (21, 14, 12, 9, and 8 items) within a single cohort of Mexican university students. Confirmatory factor analyses for ordinal data were used to test three-factor, hierarchical, and bifactor models. Additional evidence included hierarchical bifactor indices (ECV, *ω*H, PUC), reliability, score equivalence with the DASS-21, convergent validity with coping strategies, criterion-related validity based on recent functional impairment and self-reported prior psychological diagnosis, and measurement invariance by sex for the best-performing version.

**Results:**

All versions showed adequate structural performance and external associations consistent with theoretical expectations. However, bifactor analyses indicated that the general distress factor became increasingly dominant as the number of items decreased, particularly in the DASS-9 and DASS-8, reducing discrimination among the specific dimensions. In contrast, the DASS-12 showed the most favorable balance between brevity, dimensional differentiation, and external validity, and also demonstrated scalar invariance by sex.

**Conclusion:**

Although all evaluated versions were useful for screening general emotional distress, the DASS-12 emerged as the most balanced short form for Mexican university students, combining operational efficiency with adequate preservation of the three-factor structure and comparability across sex.

## 1 Introduction

Early identification of depression, anxiety, and stress symptoms in university students has become increasingly important for institutional mental health screening and well-being programs. In these settings, instruments must be brief enough for large-scale administration while preserving psychometric quality and interpretability. The Depression, Anxiety and Stress Scales-21 (DASS-21) has therefore become a widely used measure in non-clinical populations because it captures emotional distress through a stable three-factor structure and can be efficiently administered in educational contexts (Lovibond and Lovibond, 1995). However, its length may still represent an operational constraint when included in broad assessment batteries, which has motivated the development of abbreviated versions such as the DASS-14, DASS-12, DASS-9, and DASS-8 (Ali et al., 2021; Lee et al., 2019; Wise et al., 2017; Yusoff, 2013).

### 1.1 Background

Internationally, the DASS has been widely used in both clinical and non-clinical populations, showing robust psychometric properties and broad cross-cultural applicability. Previous studies have supported its factorial validity, internal consistency, and sensitivity to varying levels of emotional distress in community, student, and clinical samples (Antony et al., 1998; Henry and Crawford, 2005). In recent years, research has further reinforced its utility for large-scale screening and has examined its dimensional structure in greater depth, particularly through hierarchical and bifactor models that capture the coexistence of a general distress factor alongside more specific symptom domains (Domínguez-Lara, 2024). At the same time, studies from different cultural contexts have increasingly evaluated abbreviated forms of the instrument, highlighting both their operational efficiency and the tensions they introduce between brevity and dimensional precision (Ali et al., 2021; Lee et al., 2019).

This distinction is also theoretically meaningful. From the perspective of the tripartite model, depression and anxiety share a broad component of general negative affect, while also retaining more specific features, such as low positive affect in depression and physiological hyperarousal in anxiety (Clark and Watson, 1991). In turn, the DASS framework extends this logic by incorporating stress as a related but distinguishable domain characterized by tension, irritability, and difficulty relaxing (Lovibond and Lovibond, 1995). Therefore, the evaluation of abbreviated DASS versions is not only a matter of operational efficiency, but also of whether reduced forms preserve theoretically meaningful differentiation within emotional distress rather than collapsing into a largely undifferentiated general factor.

The function of the DASS also varies according to the context of application. In clinical settings, the instrument is typically used to quantify symptom severity and to complement broader clinical assessment, where detailed differentiation among depression, anxiety, and stress may be especially relevant. In contrast, in non-clinical contexts such as university populations, DASS is often used as a screening and monitoring tool, where efficiency, scalability, and sensitivity to change become central concerns (Kessler et al., 2002; Lovibond and Lovibond, 1995; Silva Castillo et al., 2025). Compared with broader nonspecific distress screens used in population monitoring, the DASS is distinctive in attempting to represent both a general emotional distress component and three related domains within a single instrument. Under these conditions, abbreviated versions become especially attractive, but only if they retain sufficient psychometric robustness and meaningful dimensional differentiation.

Among abbreviated formats, the DASS-14 was proposed as a refinement intended to improve construct representation in briefer form, the DASS-12 sought a balanced four-item representation of each domain, the DASS-9 reduced the structure to three indicators per factor, and the DASS-8 was developed as an ultra-brief alternative for rapid screening (Ali et al., 2021; Lee et al., 2019; Wise et al., 2017; Yusoff, 2013). Although these versions have generally shown acceptable psychometric performance, contemporary psychometric literature has consistently warned that item reduction may increase the dominance of the general factor and weaken the precision and interpretability of specific subscales, particularly in ultra-short forms (Ali et al., 2021; McNeish and Wolf, 2020; Reise et al., 2013; Smith et al., 2000). Consequently, the choice of an abbreviated version should not be based solely on convenience, but on an integrated evaluation of efficiency, structural validity, dimensional differentiation, and applied usefulness.

The proliferation of abbreviated versions of the DASS has therefore generated an important practical problem. Although several abbreviated versions have shown acceptable psychometric performance when examined individually, there is still little comparative evidence to guide the selection of a specific version on the basis of brevity, dimensional structure, and external validity. As a result, researchers and practitioners often select a short form primarily for reasons of convenience rather than on the basis of integrated psychometric evidence (AERA, APA, NCME, 2014; Messick, 1995; Norton, 2007). This issue is particularly relevant because the lack of direct within-sample comparisons, the limited use of hierarchical indices to evaluate the relative contribution of the general versus specific factors, and the scarce incorporation of external criteria make it difficult to determine whether a brief form captures only global distress or preserves meaningful differentiation among depression, anxiety, and stress (Shields et al., 2021; Wang et al., 2025; Yun, 2025).

In the Mexican context, this gap is especially important. Although the DASS-21 has shown adequate psychometric properties in university and community samples, its full length may be less suitable for large-scale institutional assessment or repeated monitoring of student well-being (Cruz-Flores et al., 2024; Salinas-Muñoz et al., 2024; Rolstad et al., 2011). Recent Mexican evidence has documented the trifactorial structure of the full version, its internal consistency, and its linguistic adaptation, convergent validity, and measurement invariance by sex in university students (Cruz-Flores et al., 2024; Salinas-Muñoz et al., 2024; Silva Castillo et al., 2025). However, these studies have focused on the full instrument and have not systematically compared abbreviated versions within the same Mexican cohort. Likewise, regional psychometric work has highlighted the relevance of bifactor approaches and the performance of simplified DASS forms, but without consistently integrating functional indicators or self-reported diagnostic information in a single comparative framework (Domínguez-Lara, 2022, 2024).

A second limitation concerns the kind of validity evidence typically used to justify brief screening tools. The practical value of a screening instrument cannot be established solely from internal structure and reliability; it also depends on whether scores relate meaningfully to external criteria relevant to everyday functioning (AERA, APA, NCME, 2014; Messick, 1995). In university populations, coping strategies represent one such criterion: adaptive forms of coping, such as proactive or reflective coping, should be associated with lower emotional distress, whereas avoidant coping should be associated with higher distress (Schwarzer and Taubert, 2002). Likewise, brief indicators of recent functional interference—such as doing less than desired or stopping daily activities because of emotional or physical discomfort—provide applied evidence of the extent to which distress affects daily life (Carver et al., 1989). Self-reported prior psychological diagnoses, although not equivalent to clinical assessment, may further help determine whether a brief form discriminates between groups with different levels of risk (Santos et al., 2021). These external criteria strengthen both the validity argument and the applied relevance of abbreviated screening instruments.

### 1.2 Purpose, research questions, and contribution

Against this background, the purpose of the present study was to compare five versions of the DASS (21, 14, 12, 9, and 8 items) within a single cohort of Mexican university students in order to determine which version offers the best balance between efficiency, dimensional differentiation, and applied utility for emotional distress screening.

To address this purpose, the study was guided by the following research questions:

RQ1. Which DASS version shows the most adequate factorial performance when evaluated under three-factor, hierarchical, and bifactor models?

RQ2. To what extent do the abbreviated versions preserve an interpretable balance between the general emotional distress factor and the specific depression, anxiety, and stress factors?

RQ3. Do the abbreviated versions retain functional equivalence with the DASS-21 and preserve expected associations with coping, functional impairment, and self-reported prior psychological diagnosis?

RQ4. Does the best-performing abbreviated version demonstrate measurement invariance by sex, thereby supporting valid group comparisons?

Guided by previous psychometric research and by the distinction between shared and specific components of emotional distress, we expected that all versions would show acceptable overall fit, that bifactor models would provide the best representation of the data, and that shorter versions would increasingly concentrate variance in a general distress factor at the expense of specific dimensional differentiation. More specifically, we expected the DASS-12 to provide the most favorable balance between brevity and preservation of interpretable subscale structure (Clark and Watson, 1991; Domínguez-Lara, 2022, 2024; Shields et al., 2021).

This study makes three contributions. First, to our knowledge, it provides the first within-sample comparison of the DASS-21, DASS-14, DASS-12, DASS-9, and DASS-8 in a Mexican university cohort, addressing an important gap in the local literature (Cruz-Flores et al., 2024; Salinas-Muñoz et al., 2024; Silva Castillo et al., 2025). Second, it integrates international and local evidence with competing latent structural models, hierarchical bifactor indices, and external validity indicators, thereby moving beyond isolated evidence based only on model fit or internal consistency (AERA, APA, NCME, 2014; Messick, 1995). Third, it evaluates whether the most efficient abbreviated version also retains practical interpretability and measurement invariance by sex, generating evidence that is directly useful for institutional screening and student well-being assessment. In this way, the study seeks not only to identify a shorter DASS version, but also to clarify which abbreviated form constitutes the most psychometrically robust, theoretically coherent, and practically informative option for use in Mexican university populations.

## 2 Materials and methods

### 2.1 Design and relation to prior study

The data analyzed in this study derive from the same cohort used in the Mexican validation of the DASS-21 (Silva Castillo et al., 2025). The present manuscript constitutes a set of secondary analyses with a distinct focus: the psychometric comparison of abbreviated versions of the DASS (DASS-12, DASS-9, and DASS-8) relative to the full DASS-21, as well as the examination of external validity indicators (coping strategies and functional impairment/self-reported diagnosis). These objectives were not addressed in the original publication (Silva Castillo et al., 2025). Accordingly, previously established decisions regarding linguistic adaptation and the estimation framework for ordinal items were retained.

### 2.2 Participants

A total of *N* = 1,251 first-year undergraduate students participated in the study, drawn from the same cohort described in Silva Castillo et al. (2025). Participants were enrolled at a private university in Mexico and represented diverse academic disciplines. The mean age was 18.65 years (SD = 1.40). Because of a data entry error, 17.5% of age values were missing. The sample was approximately balanced by sex (50.3% men, 48.5% women; remaining categories <1.5%).

Inclusion criteria were: (a) enrollment as a first-year undergraduate student during the 2024–2025 academic cycle, (b) provision of informed consent, (c) sufficient Spanish language proficiency to comprehend the assessment materials, (d) basic digital literacy skills to complete an online questionnaire, and (e) access to university computer facilities where the assessment was administered under supervised conditions. Exclusion criteria included incomplete responses or failure to provide consent.

Participants were assessed within a structured institutional context during their first academic year, as part of a broader evaluation process of student well-being. In terms of self-reported impact, 25.4% of participants indicated accomplishing less than they would have liked, whereas 12.6% reported discontinuing activities due to emotional distress.

### 2.3 Procedure

Data were collected at a single site through a digital administration conducted in university computer laboratories between August 12 and August 18, 2024. The assessment was conducted under standardized conditions, following the same protocol described in the original Mexican validation of the DASS-21 (Silva Castillo et al., 2025).

All instruments were administered via SurveyMonkey®, with randomization of item order and the sociodemographic block. Prior to participation, students received standardized instructions delivered by trained research assistants following a structured administration script. All assistants were previously trained to ensure consistency in the explanation of procedures, resolution of participant questions, and management of the assessment process.

Participants completed the assessment individually in supervised university computer laboratories. This controlled setting reduced the likelihood of careless responding, multiple submissions, or environmental distractions (Shadish et al., 2002), while also ensuring that access to the survey link was mediated exclusively by the research team. The standardized laboratory conditions enhanced the uniformity and comparability of responses across participants (AERA, APA, NCME, 2014; Gosling et al., 2004). Responses were downloaded using anonymous identifiers to ensure data pseudonymization.

### 2.4 Instruments

#### 2.4.1 DASS-21 and abbreviated versions

The study used the recently validated Mexican version of the Depression, Anxiety and Stress Scales-21 (DASS-21), which retains the trifactorial structure originally proposed by Lovibond and Lovibond (1995) and has shown evidence of scalar measurement invariance by sex in university populations. This adaptation preserves the conceptual content of the original instrument, with minimal linguistic adjustments to Mexican Spanish.

Based on this version, four abbreviated forms commonly reported in the literature were evaluated: the DASS-14 (Wise et al., 2017), DASS-12 (Lee et al., 2019), DASS-9 (Yusoff, 2013), and DASS-8 (Ali et al., 2021). All abbreviated versions preserve semantic and conceptual equivalence with the full instrument and correspond to configurations supported by international and regional studies, including comparative analyses by Domínguez-Lara (2022, 2024). These versions allow examination of the impact of item reduction on factorial structure, the relative weight of the general factor, and the retention of specific dimensions.

A detailed mapping of each version—including the item-by-item allocation from the DASS-21 to the DASS-14, DASS-12, DASS-9, and DASS-8—is provided in Supplementary Table S1. This Supplementary material ensures full transparency regarding item selection and allows precise replication of all abbreviated versions evaluated in the present study.

In the main text, only a summary of the composition of each version is presented (Table 1), indicating the total number of items and their distribution across subscales (depression, anxiety, and stress).

**Table 1:** Composition of DASS versions.

Version	References	Total items (*n*)	Depression items (*n*)	Anxiety items (*n*)	Stress items (*n*)
DASS-21	Antony et al. (1998)	21	7 (3, 5, 10, 13, 16, 17, 21)	7 (1, 2, 4, 6, 18, 19, 20)	7 (7, 8, 9, 11, 12, 14, 15)
DASS-14	Wise et al. (2017)	14	5 (3, 10, 16, 17, 21)	3 (4, 7, 19)	6 (1, 6, 11, 12, 14, 18)
DASS-12	Lee et al. (2019)	12	4 (5, 10, 16, 17)	4 (7, 9, 15, 20)	4 (6, 11, 12, 14)
DASS-9	Yusoff (2013)	9	3 (5, 10, 16)	3 (7, 9, 15)	3 (6, 11, 14)
DASS-8	Ali et al. (2021)	8	3 (10, 13, 16)	3 (9, 15, 20)	2 (8, 12)

#### 2.4.2 Proactive Coping Inventory—Adapted Version (PCI-VA)

Coping strategies were assessed using the Proactive Coping Inventory—Adapted Version (PCI-VA), a Mexican adaptation of the original Proactive Coping Inventory (PCI) developed by the research team for use with university students in accordance with the adaptation guidelines proposed by Greenglass et al. (1999). The PCI is a multidimensional self-report instrument designed to assess cognitive and behavioral strategies involved in the anticipatory and reactive management of stressors. Consistent with the process-oriented framework underlying the original instrument, the adapted version distinguishes among action-oriented, planning-based, support-related, and avoidance-oriented coping responses.

The PCI-VA comprises five dimensions: Instrumental/Emotional Support Seeking, Reflective/Preventive Coping, Proactive Coping, Avoidant Coping, and Strategic Planning. Items are rated on a 4-point Likert-type scale. Although the instrument yields both subscale and total scores, the present study focused on the five subscale scores, which were computed by summing the corresponding item responses. Higher scores indicate greater use of the coping strategy represented by each dimension. In the present study, these subscale scores were used as external criteria to examine the convergent validity of the full and abbreviated versions of the DASS.

Psychometric evidence for the PCI-VA has been reported in the same university cohort analyzed in the present study (Silva Castillo et al., 2025). Internal consistency was satisfactory across subscales, with Cronbach’s alpha coefficients ranging from 0.70 to 0.90 and McDonald’s omega coefficients ranging from 0.75 to 0.90; bootstrap 95% confidence intervals further supported the stability of these estimates. Subscale-specific reliability coefficients were as follows: Strategic Planning (*α* = 0.70, *ω* = 0.75), Avoidant Coping (*α* = 0.77, *ω* = 0.79), Instrumental/Emotional Support Seeking (*α* = 0.90, *ω* = 0.90), Proactive Coping (*α* = 0.87, *ω* = 0.88), and Reflective/Preventive Coping (*α* = 0.89, *ω* = 0.89). Structural validity was also supported by exploratory and confirmatory factor analyses, which showed satisfactory model fit (*χ*^2^ = 2051.64, df = 892, *p* < 0.001; RMSEA = 0.060; CFI = 0.978; TLI = 0.977; GFI = 0.969). In sum, these findings support the use of the PCI-VA as a psychometrically adequate multidimensional measure of coping in Mexican university students.

#### 2.4.3 Sociodemographic questionnaire

A brief sociodemographic questionnaire was included to assess age, sex, and academic semester, as well as self-reported indicators of recent functioning. Physical activity frequency was recorded using a single item with five ordinal response categories: daily, more than three times per week, two or three times per week, once per week, and never.

In addition, indicators of functional impairment during the previous week were assessed across two domains: physical functioning (did less than desired; had to stop daily activities) and emotional functioning (did less than desired; had to stop daily activities). Responses were dichotomous (yes/no). These indicators were used as external criteria for known-groups validity analyses.

### 2.5 Ethics and transparency

The study was reviewed and approved by the Research Ethics Committee (Approval No. 072-CEI08-2024, August 2024), in accordance with the principles of the Declaration of Helsinki (World Medical Association, 2013) and the Regulations of the General Health Law on Health Research (Secretaría de Salud, 2014).

All participants were individually informed about the objectives and procedures of the study and provided written informed consent, including explicit authorization for the use of their data for research purposes. Participation was entirely voluntary, no financial or academic incentives were provided, and participants had the right to withdraw at any time without consequences.

Data handling procedures were conducted in accordance with applicable local regulations on confidentiality and data protection. Confidentiality was protected through data pseudonymization, whereby direct identifiers were replaced with randomly generated codes. All records were securely stored, and access was restricted exclusively to the research team.

The data analyzed in this study derive from the same cohort reported in the Mexican validation of the DASS-21 (Silva Castillo et al., 2025). The present manuscript constitutes a set of secondary analyses with a distinct purpose, focused on the psychometric comparison between abbreviated versions of the DASS (DASS-12, DASS-9, and DASS-8) and the full version, as well as on the evaluation of external validity using coping strategies and indicators of functional impairment. These objectives were not addressed in the original publication. Previously established decisions regarding linguistic adaptation, data cleaning criteria, and the estimation framework for ordinal data were retained. In addition, the present study incorporates new sources of evidence not included in the original report, thereby supporting an integrated comparative and applied psychometric evaluation.

All theoretical and methodological decisions were made independently by the research team, and no conflicts of interest are declared.

### 2.6 Data analysis

#### 2.6.1 Data preparation and preliminary assumptions

All analyses were conducted in R (version 4.5.1; R Core Team, 2024), using RStudio (Posit Team, 2025) and the packages lavaan (Rosseel et al., 2025), semTools (Jorgensen et al., 2025), and psych (Revelle, 2025) for psychometric analyses, as well as Tidyverse for data management and visualization (Wickham et al., 2019). All procedures followed the Standards for Educational and Psychological Testing (AERA, APA, NCME, 2014), the EFPA model for test evaluation (European Federation of Psychologists’ Associations, 2025), and the STROBE guidelines for observational studies (von Elm et al., 2007). The significance level was set at *α* = 0.05, and 95% confidence intervals were reported when applicable.

Data preparation involved an initial examination of response completeness, followed by the identification of multivariate outliers using Mahalanobis distance. Univariate item distributions were then inspected, considering values of skewness below |2| and kurtosis below |7| as indicative of acceptable departures from normality. Sample adequacy was evaluated using the Kaiser–Meyer–Olkin (KMO) index, with values above 0.80 interpreted as evidence of sufficient sampling adequacy. In addition, sample size adequacy for confirmatory factor analysis and bifactor modeling was evaluated based on absolute N requirements and subject-to-parameter ratios. The total sample (*N* = 1,251 participants) exceeded the minimum threshold of 200–300 participants recommended for stable CFA solutions with ordinal data (Kyriazos, 2018).

For bifactor models, which entail greater parameter complexity, the sample provided a subject-to-free-parameter ratio of approximately 19:1, surpassing the conservative guideline of 5:1–10:1 (Comrey and Lee, 1992; MacCallum et al., 1999). This indicates a robust basis for parameter estimation in complex latent variable models.

Importantly, because the bifactor specification represents the most parameter-intensive model evaluated in this study, its adequate estimation implies that simpler models (i.e., three-factor and hierarchical structures) are also sufficiently supported by the available sample size. Consequently, the sample provides adequate statistical power not only for stable parameter estimation, but also for meaningful comparison between alternative structural models across DASS versions.

Given the ordinal nature of the DASS items, all factor analyses were estimated using polychoric correlation matrices, consistently applying the WLSMV/DWLS estimator. This estimator was selected because it provides robust parameter estimates and standard errors for ordinal indicators and performs adequately under conditions of non-normality, which are common in Likert-type data (Brown, 2015; Flora and Curran, 2004).

#### 2.6.2 Evaluation of internal structure

The internal structure of the DASS was examined using confirmatory factor analysis (CFA) across five versions of the instrument (21, 14, 12, 9, and 8 items). For each version, three theoretically grounded specifications were estimated: an oblique three-factor model reflecting the traditional dimensions of stress, anxiety, and depression; a hierarchical model comprising three first-order factors and a second-order general factor; and a bifactor model consisting of a general emotional distress factor alongside three orthogonal specific factors.

All factor loadings were constrained to their corresponding theoretical factors, strictly replicating the configurations proposed in previous studies (Domínguez-Lara, 2022, 2024). No *post hoc* modifications, residual correlations, or cross-loadings were introduced, in order to preserve comparability across versions. Model fit was evaluated using the *χ*^2^ statistic, CFI, TLI, RMSEA (with 90% confidence intervals), and SRMR. Acceptable model fit was defined by CFI and TLI values ≥0.95, RMSEA ≤0.06, and SRMR ≤0.08, and solutions were considered practically equivalent when differences between models did not exceed ΔCFI = 0.010 and ΔRMSEA = 0.015 (Chen, 2007).

As a sensitivity analysis, exploratory structural equation models (ESEM) were estimated to examine the potential presence of cross-loadings; however, substantive interpretation was based exclusively on the confirmatory models.

#### 2.6.3 Bifactor dimensionality and general factor strength

To characterize the dimensionality of the bifactor solutions, a set of hierarchical indices proposed by Rodríguez et al. (2016) was computed, including Explained Common Variance (ECV), hierarchical omega (*ω*H), and the Proportion of Uncontaminated Correlations (PUC). ECV represents the proportion of common variance attributable to the general factor, *ω*H estimates the proportion of reliable variance in total scores explained by the general factor, and PUC reflects the proportion of correlations among items that are influenced only by the general factor.

These indices were used to evaluate the relative magnitude of the general factor in relation to the specific factors. Values approaching ECV ≥ 0.70 and *ω*H ≥ 0.80 were interpreted as evidence of essential unidimensionality, whereas intermediate values were taken to indicate hierarchical configurations in which a dominant general factor coexists with residual differentiation across domains. Together, these indices provide complementary information about the extent to which total scores can be interpreted as reflecting a primarily general versus multidimensional construct.

#### 2.6.4 Reliability

Reliability was examined using Cronbach’s alpha and McDonald’s omega, both reported with 95% confidence intervals, for subscale scores as well as for total scores. For bifactor models, hierarchical omega (*ω*H) and Explained Common Variance (ECV) were additionally reported in order to estimate the proportion of variance attributable to the general factor. Differences of 0.05 or smaller between versions were interpreted as being of small magnitude, indicating negligible practical differences in reliability estimates.

#### 2.6.5 Functional equivalence across versions

Functional equivalence between the DASS-21 and the abbreviated versions was evaluated using Pearson or Spearman correlations, depending on score distributions, between total scores and corresponding subscale scores across forms. An abbreviated version was considered to provide information essentially equivalent to the full scale when the correlation between total scores reached values of *r* ≥ 0.95.

These associations were interpreted as evidence of functional equivalence rather than as indicators of independent validity, given the partial overlap of items between the abbreviated and full versions.

#### 2.6.6 Convergent validity

Convergent validity was examined using Spearman’s rank-order correlations (*ρ*) between DASS scores across all five versions and the dimensions of the Proactive Coping Inventory—Adapted Version (PCI-VA). Negative associations were expected with adaptive coping strategies—including proactive, reflective/preventive, instrumental/emotional support seeking, and strategic planning—whereas positive associations were expected with avoidant coping.

To control the familywise Type I error rate, all *p* values were adjusted using the Holm–Bonferroni correction.

#### 2.6.7 Criterion-related validity and known-groups analyses

Criterion-related validity was examined through known-groups comparisons based on sex and recent functional impairment. Depending on distributional assumptions and the homogeneity of variances, group differences were tested using Welch’s *t* test, the Mann–Whitney *U* test, or the Kruskal–Wallis test, as appropriate. Effect sizes were expressed using Cohen’s d or the rank-biserial correlation (*r*), and were reported together with their 95% confidence intervals.

#### 2.6.8 Multigroup factorial invariance

Based on its overall psychometric performance, the DASS-12 was selected for multigroup factorial invariance testing by sex. A hierarchical sequence of configural, metric, and scalar models was estimated using the WLSMV estimator. Decisions regarding invariance were guided by changes in fit indices, specifically ΔCFI ≤0.010 and ΔRMSEA ≤0.015. The attainment of scalar invariance was interpreted as evidence that latent mean comparisons between men and women are valid.

## 3 Results

### 3.1 Descriptive statistics and reliability

The sample comprised 1,251 first-year university students (M age = 18.65, SD = 1.40) with a balanced sex distribution, providing a stable basis for subsequent psychometric analyses. Detailed sociodemographic characteristics are presented in Table 2.

**Table 2:** Sociodemographic characteristics of the sample.

Variable	Category	*n*	%
Gender identity	Man	629	50.3
	Woman	607	48.5
	Non-binary	5	0.4
	Other	1	0.1
	Prefer not to say	9	0.7
Marital status	Single	1,018	81.4
	In a relationship	210	16.8
	Prefer not to answer	20	1.6
	Other	3	0.2
Self-reported condition	Yes	154	12.3
	No	1,061	84.8
	Prefer not to answer	36	2.9

Percentages are based on the total sample (*N* = 1,251). Categories with frequencies below 1% are retained for completeness. Self-reported condition refers to prior psychological or medical diagnosis as reported by participants.

Item-level distributions for the DASS were within acceptable ranges of skewness and kurtosis for ordinal data, indicating no substantial departures from distributional assumptions (see Supplementary Table S2). In addition, sampling adequacy was high (KMO > 0.80), supporting the suitability of the data for latent variable modeling.

These results indicate that the data met the necessary assumptions for reliable estimation of the structural models evaluated in the following analyses.

### 3.2 Reliability of the DASS versions

As shown in Table 3, all DASS versions demonstrated adequate internal consistency across most dimensions, with McDonald’s omega coefficients generally exceeding the conventional threshold (*ω* ≥ 0.70). This pattern indicates that item reduction did not substantially compromise the overall reliability of the instrument at the total-score level.

**Table 3:** Omega reliability coefficients by dimension across DASS versions.

Version	Depression [95% CI]	Anxiety [95% CI]	Stress [95% CI]
DASS-21	0.85 [0.84–0.87]	0.85 [0.83–0.86]	0.88 [0.87–0.89]
DASS-14	0.81 [0.80–0.83]	0.79 [0.77–0.81]	0.86 [0.84–0.87]
DASS-12	0.77 [0.75–0.79]	0.80 [0.78–0.82]	0.78 [0.77–0.81]
DASS-9	0.67 [0.64–0.70]	0.72 [0.70–0.75]	0.71 [0.68–0.73]
DASS-8	0.76 [0.73–0.78]	0.78 [0.75–0.80]	—

Values correspond to McDonald’s omega (*ω*) coefficients with 95% confidence intervals. Values ≥0.70 are typically considered acceptable for research purposes. The Stress subscale in the DASS-8 could not be reliably estimated due to the insufficient number of items (two-item factor).

However, two domain-specific limitations emerged in the shorter versions. First, the Depression subscale of the DASS-9 showed a slightly lower coefficient (*ω* = 0.67), suggesting reduced precision in capturing this domain with only three items. Second, reliability for the Stress subscale in the DASS-8 could not be estimated due to its two-item composition, which is insufficient for stable reliability estimation.

Collectively, these findings suggest that although ultra-brief versions preserve acceptable overall reliability, the costs of abbreviation are more evident at the subscale level, where reduced item coverage may limit the stability and interpretability of specific dimensions.

### 3.3 Structural model fit

As summarized in Table 4, all DASS versions exhibited excellent overall model fit. CFI and TLI values were consistently high (≥0.99), and approximation errors remained low, indicating that all model specifications adequately reproduced the observed covariance structure. Full results for the three-factor, hierarchical, and bifactor models are reported in Supplementary Table S3.

**Table 4:** Best-fitting model for each DASS version (*N* = 1,251).

Version	Model	CFI	TLI	RMSEA [90% CI]	SRMR
DASS-21	Bifactor	0.997	0.996	0.022 [0.017–0.027]	0.037
DASS-14	Bifactor	0.996	0.994	0.029 [0.022–0.036]	0.037
DASS-12	Bifactor	0.998	0.997	0.019 [0.006–0.029]	0.028
DASS-9	Bifactor	0.996	0.992	0.033 [0.020–0.046]	0.031
DASS-8	Bifactor	0.997	0.994	0.033 [0.017–0.049]	0.028

CFI, Comparative Fit Index; TLI, Tucker–Lewis Index; RMSEA, Root Mean Square Error of Approximation; SRMR, Standardized Root Mean Square Residual. Values reflect excellent model fit across all versions (CFI/TLI ≥0.95; RMSEA ≤0.06; SRMR ≤0.08). Full fit indices for three-factor, hierarchical, and bifactor models are reported in Supplementary Table S3.

Across all versions, the bifactor model provided the best representation of the data. Although improvements in fit relative to the three-factor and hierarchical models were modest (ΔCFI ≤0.010; ΔRMSEA ≤0.015), they were consistent, supporting the presence of a general emotional distress factor that accounts for shared variance across items while retaining domain-specific components.

Notably, differences between models were not large enough to justify interpretation based on fit alone. This suggests that model selection should consider not only statistical fit but also the substantive meaning of the latent structure, particularly in the context of abbreviated measures.

Among the abbreviated versions, the DASS-12 showed the most favorable balance between model fit and parsimony. Although the DASS-9 and DASS-8 also achieved good overall fit, their bifactor solutions indicated a stronger concentration of variance in the general factor, suggesting reduced differentiation among the specific dimensions. These findings point to a systematic trade-off between brevity and dimensional resolution, with shorter versions increasingly favoring a general distress representation over a multidimensional structure.

### 3.4 Bifactorial dimensionality and general factor weight

**Figure 1:**
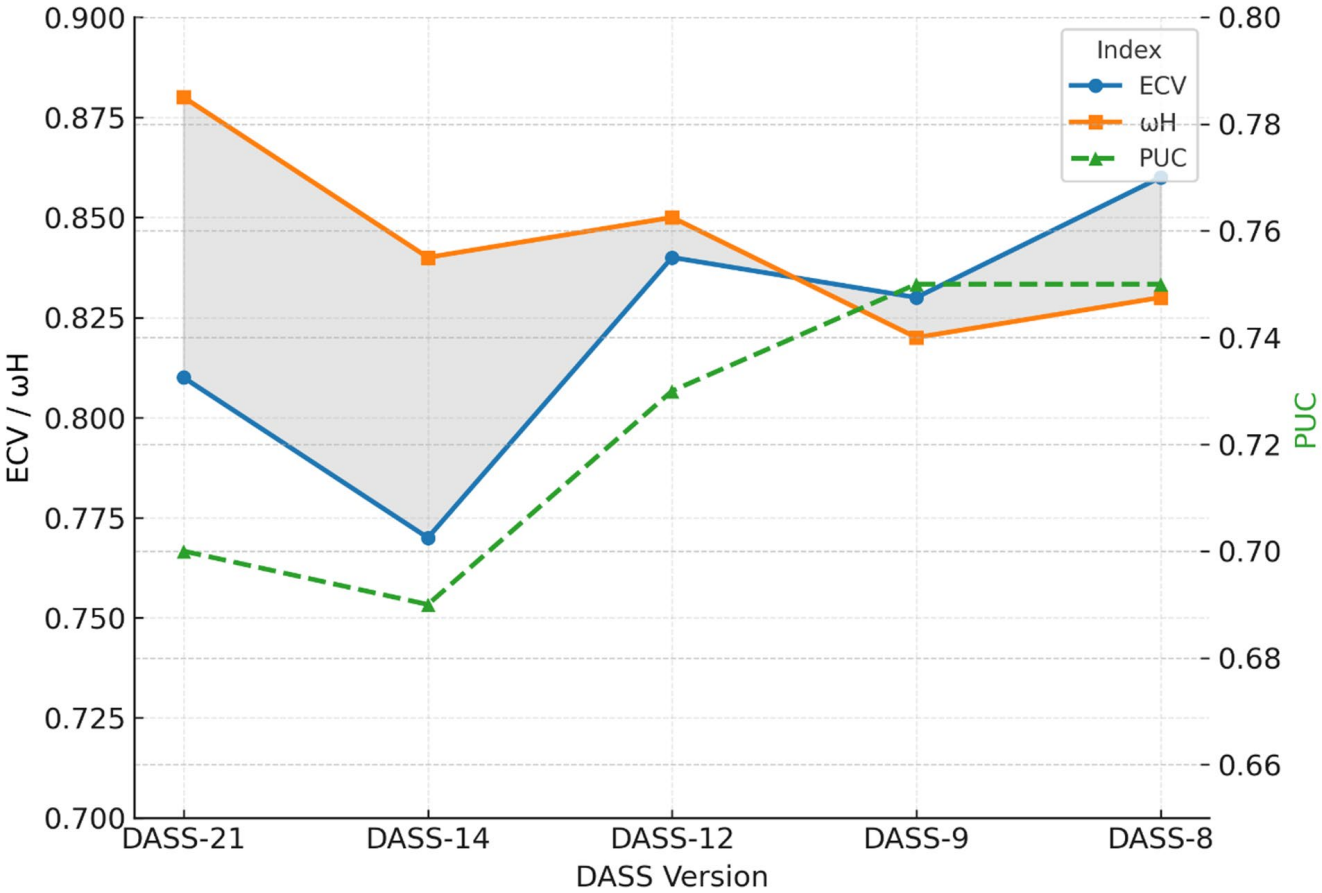
Bifactor indices (ECV, *ω*H, and PUC) across DASS versions. ECV, Explained Common Variance; *ω*H, hierarchical omega; PUC, Proportion of Uncontaminated Correlations. Values illustrate the increasing dominance of the general factor as the number of items decreases.

To facilitate interpretation, ECV represents the proportion of common variance explained by the general factor, *ωH* reflects the proportion of reliable variance attributable to the general factor, and *PUC* indicates the proportion of correlations primarily influenced by the general factor. Figure 1 displays the evolution of these bifactor indices across the five DASS versions. Detailed bifactor reliability and dimensionality indices supporting these interpretations are reported in Supplementary Table S5a.

Overall, the results indicate a systematic increase in the dominance of the general factor as the number of items decreases. Specifically, ECV and *ωH* values increased in the shorter versions, whereas PUC values showed a slight downward trend. This pattern reflects a progressive concentration of common variance in the general factor, accompanied by a relative reduction in the contribution of specific factors.

Within this continuum, the DASS-12 occupied an intermediate position. It showed elevated ECV and *ωH* values, supporting the presence of a robust general factor, while maintaining PUC levels consistent with meaningful residual differentiation among stress, anxiety, and depression.

In contrast, the DASS-9 and DASS-8 displayed patterns consistent with *essential unidimensionality*, indicating that the general factor accounted for most of the reliable variance and that the specific factors contributed comparatively little unique information. In practical terms, this suggests that these ultra-brief versions function primarily as indicators of overall emotional distress rather than as multidimensional measures.

### 3.5 Functional equivalence across versions

The abbreviated DASS versions showed strong correspondence with the DASS-21, as reflected in high correlations between total and subscale scores (*ρ* = 0.87–0.99; see Supplementary Table S6). This pattern indicates that the essential information captured by the full instrument is largely preserved in the shorter forms.

Of relevance, these associations should be interpreted as evidence of functional equivalence rather than as indicators of independent validity, given the partial overlap of items across versions. In this context, the results support the use of abbreviated forms as efficient proxies for the DASS-21 in screening applications, while acknowledging that they do not constitute fully independent measures of emotional distress.

### 3.6 Convergent validity with coping (PCI-VA)

Correlations between DASS scores and the dimensions of the Proactive Coping Inventory—Adapted Version (PCI-VA) followed the theoretically expected pattern (Table 5). Across all versions, higher levels of emotional distress were associated with lower use of adaptive coping strategies and greater reliance on avoidant coping, consistent with established models of stress and coping.

**Table 5:** Correlations between total DASS scores (by version) and PCI-VA dimensions.

Version	Support	Reflective/preventive	Proactive	Avoidant	Strategic
21	−0.11***	−0.06*	−0.21***	0.42***	−0.13***
14	−0.12***	−0.06*	−0.20***	0.41***	−0.13***
12	−0.12***	−0.06*	−0.21***	0.42***	−0.14***
9	−0.10***	−0.05	−0.21***	0.40***	−0.12***
8	−0.10***	−0.05	−0.20***	0.40***	−0.14***

Values are Spearman’s *ρ* coefficients. Negative correlations with adaptive coping (support, reflective/preventive, proactive, strategic) and positive correlations with avoidant coping are theoretically expected. All *p* values were adjusted using the Holm–Bonferroni correction. *p < 0.05, **p < 0.01, ***p < 0.001.

Importantly, differences between the abbreviated versions and the DASS-21 were negligible (|Δ*ρ*| < 0.02), indicating that item reduction did not meaningfully alter the pattern or magnitude of external associations. This stability suggests that the convergent validity of the instrument is largely preserved across versions, despite differences in length.

Among the abbreviated versions, the DASS-12 again showed the most favorable balance between parsimony and retention of theoretically coherent external relations, supporting its use as a brief yet valid indicator of emotional distress in relation to coping processes.

### 3.7 Criterion-related validity and known-groups analyses

As shown in Table 6, effect sizes across DASS versions were highly comparable as a function of sex and recent functional impairment indicators. Overall, the pattern of group differences was stable across the full and abbreviated forms.

**Table 6:** Cross-version effect sizes (total DASS) with positive direction conventions.

Version	Sex *g* (Women > Men) [95% CI]	Impairment 1 *r* (Yes > No) [95% CI]	Impairment 2 *r* (Yes > No) [95% CI]
DASS-21	0.31 [0.20, 0.43]	0.47 [0.44, 0.50]	0.53 [0.50, 0.56]
DASS-14	0.28 [0.17, 0.40]	0.45 [0.42, 0.48]	0.51 [0.48, 0.54]
DASS-12	0.31 [0.20, 0.43]	0.45 [0.42, 0.48]	0.51 [0.48, 0.54]
DASS-9	0.29 [0.18, 0.40]	0.43 [0.40, 0.46]	0.49 [0.46, 0.52]
DASS-8	0.32 [0.20, 0.43]	0.45 [0.42, 0.48]	0.51 [0.48, 0.54]

*g* = Hedges’ *g*; *r* = rank-biserial correlation. Positive values indicate higher scores in women (sex comparison) or in participants reporting impairment (Yes > No). Effect sizes are interpreted as small (≈ 0.20), moderate (≈ 0.50), and large (≈ 0.80). Impairment 1 = did less than desired; impairment 2 = stopped daily activities.

Sex differences were consistently small in magnitude, with women reporting slightly higher levels of emotional distress across all versions. This pattern aligns with previous evidence documenting modest but systematic sex differences in internalizing symptoms.

In contrast, functional impairment indicators showed small-to-moderate effects. Participants who reported doing less than desired or having to stop daily activities due to physical or emotional discomfort consistently exhibited higher distress scores across all DASS versions. Confidence intervals for effect sizes overlapped substantially across versions, indicating that the magnitude of group differences remained stable regardless of instrument length.

In sum, these findings suggest that item reduction did not meaningfully affect the instrument’s ability to discriminate between relevant groups. In practical terms, abbreviated versions retain comparable sensitivity to functional impairment, supporting their use in applied screening contexts.

### 3.8 Multigroup factorial invariance of the DASS-12 by sex

Measurement invariance of the DASS-12 across sex was evaluated through a sequence of configural, metric, and scalar models. The configural model showed adequate fit, indicating that the underlying three-factor structure is comparable for men and women.

When equality constraints were imposed on factor loadings and thresholds, changes in fit indices were minimal (ΔCFI ≤0.001; ΔRMSEA ≤0.006) and remained well within commonly accepted criteria for invariance.

These results support full scalar invariance of the DASS-12, indicating that both the measurement structure and item thresholds operate equivalently across groups. Consequently, comparisons of latent means between men and women can be interpreted as psychometrically valid (see Table 7).

**Table 7:** Measurement invariance analysis of the DASS-12 by sex (*N* = 1,236).

Model	*χ* ^2^	df	CFI	RMSEA	SRMR	ΔCFI	ΔRMSEA
Configural	381.92	02	0.992	0.067	0.049	—	—
Metric (equal loadings)	405.32	11	0.991	0.066	0.051	0.000	−0.001
Scalar (equal loadings + thresholds)	396.54	23	0.992	0.060	0.049	0.001	−0.006

CFI, Comparative Fit Index; RMSEA, Root Mean Square Error of Approximation; SRMR, Standardized Root Mean Square Residual. Invariance decisions were based on ΔCFI ≤0.010 and ΔRMSEA ≤0.015. Results support configural, metric, and scalar invariance across sex.

## 4 Discussion

The present study was motivated by a practical problem identified in the Introduction: although several abbreviated versions of the DASS are available, there is still limited within-sample evidence to guide the selection of the version that best balances brevity, dimensional differentiation, and applied usefulness in Mexican university students. The adequacy of the sample, the stability of item distributions, and the use of robust estimators for ordinal indicators support the interpretation that the differences observed across versions reflect substantive psychometric properties rather than estimation artifacts (Brown, 2015; Flora and Curran, 2004). To strengthen coherence with the aims of the study, the discussion is organized around the four research questions.

### 4.1 Overview of the main findings

The central conclusion of this study is that all five versions of the instrument were psychometrically usable, but they were not equivalent in what they preserved from the original DASS framework. All versions showed acceptable structural performance, and bifactor models consistently provided the best overall representation of the data.

At the same time, item reduction was associated with a systematic shift in dimensional structure, with the general distress factor becoming progressively more dominant and the specific depression, anxiety, and stress dimensions becoming less distinct. Despite these structural changes, the pattern of external relations with coping and recent functional impairment remained largely stable, indicating that abbreviated versions retained much of the applied information captured by the DASS-21.

However, important differences emerged in the extent to which dimensional differentiation was preserved. The DASS-12 offered the clearest balance between efficiency and interpretability, while also demonstrating scalar invariance by sex.

Taken together, these findings directly address the practical problem raised in the Introduction: abbreviated forms should not be selected solely on the basis of brevity, but on their ability to retain sufficient structural and applied information to justify their intended use.

### 4.2 Factorial performance across versions (RQ1)

With respect to the first research question, all versions showed acceptable to excellent fit across confirmatory models, and the bifactor solution consistently emerged as the best-fitting representation. This pattern is consistent with earlier work indicating that the DASS reflects related but distinguishable dimensions embedded within a broader distress continuum (Antony et al., 1998; Caspi et al., 2014; Henry and Crawford, 2005; Lahey et al., 2017). It is also in line with recent Mexican evidence supporting the structural validity of the DASS-21 in university and community samples (Cruz-Flores et al., 2024; Salinas-Muñoz et al., 2024; Silva Castillo et al., 2025).

At the same time, the present findings introduce an important qualification. Although bifactor models consistently provided the best fit, the magnitude of these improvements relative to hierarchical and correlated three-factor solutions was small in practical terms. This cautions against interpreting statistical superiority as evidence of substantive superiority, particularly in instruments with conceptually overlapping content (Bonifay and Cai, 2017). In abbreviated measures, improvements in fit may partly reflect increased parsimony rather than a more accurate representation of the underlying construct. For this reason, model fit alone would not have been sufficient to guide the selection among DASS versions.

Within this broader pattern, both the DASS-14 and DASS-12 showed strong performance. However, the DASS-12 emerged as the more compelling option from a comparative perspective. Although the DASS-14 preserved slightly higher reliability in some subscales, the additional items did not translate into a clear advantage in structural interpretability or external relations. In contrast, the DASS-12 achieved excellent fit while maintaining a balanced four-item representation per domain, resulting in a more efficient and conceptually coherent abbreviated solution. This distinction is particularly relevant given that previous studies have typically evaluated abbreviated versions in isolation rather than within a unified comparative framework.

### 4.3 General distress and specific symptom domains (RQ2)

The second research question is central to the theoretical interpretation of abbreviated DASS forms. From the perspective of the tripartite model and the original DASS framework, emotional distress comprises both a shared component of negative affect and more specific symptom domains, particularly depression, anxiety, and stress (Clark and Watson, 1991; Lovibond and Lovibond, 1995). The present findings indicate that this balance is systematically altered as the scale is shortened.

As the number of items decreased, bifactor indices showed a progressive increase in the dominance of the general factor. Specifically, increases in ECV and hierarchical omega indicated that shorter versions concentrated a larger proportion of common and reliable variance in a general distress factor, while the relative contribution of the specific domains declined. This pattern is consistent with broader psychometric evidence indicating that item reduction tends to strengthen apparent unidimensionality while weakening the interpretability of subscale scores (McNeish and Wolf, 2020; Reise et al., 2013; Rodríguez et al., 2016).

This trend was most evident in the DASS-9 and DASS-8. In these ultra-brief forms, the general factor accounted for most of the reliable variance, leaving limited unique variance in the specific factors. As a result, these versions are more appropriately interpreted as indicators of overall emotional distress rather than as multidimensional instruments capable of differentiating among depression, anxiety, and stress at the subscale level. This interpretation is consistent with the broader literature on short-form development, which highlights the trade-off between brevity and content coverage (Smith et al., 2000; Widaman et al., 2011).

By contrast, the DASS-12 occupied a clear intermediate position within this continuum. Although the general factor remained strong, the specific factors retained sufficient independent variance to preserve the three-domain logic of the original instrument. Its balanced four-item-per-domain structure likely contributes to this performance, supporting its role as a psychometrically efficient yet theoretically coherent abbreviated form. This interpretation is consistent with Lee et al. (2019), who identified the DASS-12 as a viable abbreviated solution, and with Domínguez-Lara’s (2022, 2024) argument that bifactor models should be interpreted not only in terms of global fit, but also in terms of the extent to which meaningful variance remains in the specific factors.

The reliability findings reinforce this interpretation. As expected, the first costs of abbreviation emerged at the domain level rather than at the level of total scores. The Depression subscale of the DASS-9 fell below conventional reliability thresholds, and the Stress subscale of the DASS-8 could not be reliably estimated due to its two-item composition. This pattern closely mirrors the general psychometric observation that subscale precision is often the first property to deteriorate as instruments are shortened (McNeish, 2018; Reise et al., 2013).

### 4.4 Functional equivalence and external validity (RQ3)

The third research question addressed whether shorter versions preserved the applied usefulness of the DASS-21. The very high associations between the abbreviated versions and the full scale indicate strong functional equivalence, suggesting that the abbreviated versions retain most of the core information captured by the original instrument. However, because the abbreviated forms share items with the full version, these associations should be interpreted as evidence of information retention rather than as proof that the forms are fully interchangeable or independent measures (Widaman et al., 2011).

Crucially, external validity analyses underscored the practical relevance of the abbreviated versions. Across all forms, distress scores were negatively associated with adaptive coping and positively associated with avoidant coping, consistent with transactional models of stress and coping (Greenglass et al., 1999; Lazarus and Folkman, 1984). Similarly, known-groups analyses revealed highly comparable patterns across versions for sex and recent functional impairment. Participants who reported doing less than desired or stopping daily activities due to discomfort consistently showed higher distress scores, and the magnitude of these differences remained stable across forms.

Taken together, these findings indicate that shortening the instrument did not meaningfully affect its ability to recover theoretically and practically relevant external relations. This is particularly important because, as noted in the Introduction, brief screening instruments should not be evaluated solely on the basis of internal structure. From a validity perspective, the critical issue is whether shorter forms continue to behave as expected in relation to meaningful external criteria (AERA, APA, NCME, 2014).

Within this context, all abbreviated versions preserved this property to a substantial degree. However, the DASS-12 stands out in that it combines this retention of external validity with greater preservation of domain-specific differentiation than the DASS-9 and DASS-8. For institutional screening in university settings, this represents a clear advantage, as it allows for reduced respondent burden without collapsing the instrument into an almost exclusively general distress indicator.

### 4.5 Measurement invariance of the DASS-12 by sex (RQ4)

The fourth research question examined whether the best-performing abbreviated version would also support valid comparisons between men and women. The DASS-12 met configural, metric, and scalar invariance criteria, indicating that its scores can be interpreted equivalently across sex.

This finding extends prior Mexican evidence for the full DASS-21 (Silva Castillo et al., 2025) by showing that abbreviation does not necessarily compromise measurement comparability when the underlying structure is preserved. In this case, the DASS-12 maintained invariance while also achieving a favorable balance between brevity and interpretive value.

Importantly, this result is not merely technical. Small but consistent sex differences in emotional distress are commonly reported in university populations (Hyde et al., 2008), but such differences are only substantively interpretable when the measurement model operates equivalently across groups.

In this respect, the DASS-12 is particularly well suited for population monitoring and institutional well-being programs, as it combines efficiency with evidence that group comparisons are psychometrically defensible.

### 4.6 Positioning the findings within previous research on abbreviated DASS versions

One limitation of the existing literature is that abbreviated DASS versions have typically been evaluated in separate studies, under different conditions and analytic criteria, making direct comparisons difficult. The present study helps to situate these findings within a single comparative framework.

Previous research has provided support for multiple abbreviated forms under specific conditions. Wise et al. (2017) proposed the DASS-14 as an improvement in construct representation and reliability in health professionals; Lee et al. (2019) identified the DASS-12 as a balanced abbreviated version in a Korean sample; Yusoff (2013) reported acceptable psychometric performance for the DASS-9 in medical applicants; and Ali et al. (2021) validated the DASS-8 as an ultra-brief instrument for rapid screening. The present findings are broadly consistent with this literature in that none of the abbreviated forms performed poorly and all retained a degree of psychometric utility.

The contribution of the present study is therefore not to contradict prior findings, but to refine them. When these versions are examined side by side within the same Mexican university sample, and evaluated using a broader set of criteria—including model fit, bifactor indices, reliability, functional equivalence, external correlates, and measurement invariance—the DASS-12 emerges as the most balanced overall solution. Importantly, this advantage does not reflect poor performance of the DASS-14, but rather the fact that the DASS-12 achieves comparable psychometric functionality with greater parsimony and a more balanced domain composition.

Similarly, the present findings do not suggest that the DASS-9 or DASS-8 are inadequate. Instead, they indicate that their most defensible use in this population is as rapid indicators of general emotional distress, rather than as substitutes for more differentiated subscale assessment.

This synthesis is also particularly relevant for the Mexican literature. Recent local studies have provided strong support for the DASS-21 (Cruz-Flores et al., 2024; Salinas-Muñoz et al., 2024; Silva Castillo et al., 2025), but have not directly addressed which abbreviated version is most appropriate for large-scale university screening. The present findings help fill this gap by showing that the choice of version depends not only on brevity, but also on the extent to which theoretical differentiation and applied interpretability are preserved.

### 4.7 Implications for screening, assessment, and institutional practice

From an applied standpoint, the findings indicate that the selection of a DASS version should be guided by the intended purpose of assessment rather than by brevity alone. When the goal is the most comprehensive representation of depression, anxiety, and stress, the DASS-21 remains the preferred option. When the objective is to reduce administration time while preserving interpretable subscale information and supporting valid group comparisons, the DASS-12 emerges as the most appropriate choice in this sample.

Although the DASS-14 is also psychometrically acceptable, its more limited gain in efficiency and less balanced domain composition make it a less compelling alternative for routine screening. By contrast, the DASS-9 and especially the DASS-8 are more appropriately conceptualized as brief indicators of overall emotional distress, particularly in contexts where rapid assessment is prioritized over domain-level differentiation.

These distinctions are especially relevant in university settings, where screening tools are used not only to detect elevated distress but also to inform preventive interventions, triage decisions, and resource allocation. An instrument that is brief but overly dominated by a general factor may still identify at-risk individuals, yet provide limited guidance for understanding whether stress, anxiety, and depressive symptoms remain meaningfully differentiated.

In this context, the DASS-12 appears particularly well suited for institutional monitoring programs, as it offers a favorable balance between feasibility and interpretive value. This makes it a practical option for large-scale screening initiatives that require efficiency without substantially compromising the multidimensional information needed for decision-making. To facilitate applied decision-making, a practical guide for selecting DASS versions based on assessment goals, time constraints, and psychometric trade-offs is provided in Supplementary Table S7.

### 4.8 Limitations and future directions

Several limitations should be considered when interpreting these findings. First, the sample was drawn from a single private university and restricted to first-year students, which limits generalizability to other educational contexts, age groups, and clinical populations.

Second, although the sample size was sufficient for all models tested, the comparative analyses were conducted within a single dataset. Accordingly, the relative superiority of the DASS-12 should be interpreted as strong within-sample evidence rather than as a fully cross-validated conclusion. Replication in independent Mexican samples and in other cultural contexts is therefore necessary.

Third, the cross-sectional design precludes conclusions regarding temporal stability, longitudinal invariance, and sensitivity to change. This limitation is particularly relevant if abbreviated versions are to be used in repeated screening or intervention monitoring contexts.

Fourth, the external criteria were based on self-report measures. Although appropriate for applied screening purposes, these indicators are not equivalent to structured clinical interviews, clinician-rated assessments, or prospective mental health outcomes. Future research should examine whether the comparative advantages of the DASS-12 are maintained when evaluated against more diverse external criteria, in longitudinal designs, and in community and clinical samples.

## 5 Conclusion

In summary, the present study provides a within-sample comparison of the DASS-21, DASS-14, DASS-12, DASS-9, and DASS-8 in Mexican university students and suggests that the selection of an abbreviated version should not be based solely on convenience or global fit indices. Although all shortened forms were usable as screening tools, progressive item reduction was associated with a greater concentration of variance in a general distress factor and a corresponding weakening of domain differentiation.

Within this continuum, the DASS-12 emerged as the most balanced abbreviated option, combining strong structural performance, acceptable reliability, preservation of meaningful domain-specific variance, stable external relations, and scalar invariance by sex.

These findings suggest the DASS-12 as the most defensible brief alternative for emotional distress screening in university settings, particularly when efficiency is required without substantially compromising interpretive value.

## Data Availability

The data analyzed in this study is subject to the following licenses/restrictions: the dataset contains sensitive information derived from responses provided by university students and, for ethical and confidentiality reasons, cannot be made publicly available. Access to the data is restricted and may be granted only upon reasonable request to the corresponding author, subject to approval by the institutional ethics committee and compliance with applicable data protection regulations and use for academic purposes only. Requests to access these datasets should be directed to luissilva@iteso.mx.
