# Supplementary material for: Psychometric comparison of five short forms of the DASS in Mexican university students: implications for emotional distress screening
Source: Front Psychol. 2026 May 1;17:1808531. doi: 10.3389/fpsyg.2026.1808531 (PMC13184643; doi:10.3389/fpsyg.2026.1808531)
Supplement: Supplementary file 1 [file Table_1.docx]

**Index of Supplementary Materials**

**Supplementary Table S1. Conceptual mapping of DASS-21 items across abbreviated versions**. Documents the correspondence of DASS-21 items across previously published short forms (DASS-14, DASS-12, DASS-9, and DASS-8), following the original theoretical dimensions of stress, anxiety, and depression.

**Supplementary Table S2. Item-level descriptive statistics for DASS-21.** Presents means standard deviations, skewness, and kurtosis for each DASS-21 item, providing distributional evidence supporting the use of ordinal estimators in subsequent analyses.

**Supplementary Table S3**. Model fit indices for unidimensional, hierarchical, and bifactor models of the DASS Provides full fit statistics (χ², df, CFI, TLI, RMSEA, SRMR) for all tested models across DASS versions.

**Supplementary Table S4**. Item–total correlations and internal consistency diagnostics across DASS versions Reports corrected item–total correlations and McDonald’s omega if item deleted for each item across all DASS versions.

**Supplementary Table S5a.** Bifactor reliability and dimensionality indices across DASS versions. Provides explained common variance (ECV), omega hierarchical (ωH), proportion of uncontaminated correlations (PUC), and omega hierarchical subscale (ωHS), characterizing the dominance of the general distress factor.

**Supplementary Table S5b**. Reliability indices for hierarchical DASS models. Reports omega (ω) and construct replicability (H) coefficients for stress, anxiety, and depression subscales within hierarchical models.

**Supplementary Table S6**. Functional equivalence between the DASS-21 and abbreviated versions. Presents Spearman correlations between DASS-21 scores and those of abbreviated versions, evaluating the preservation of essential information across forms.

**Supplementary Table S7.** Practical guide for selecting DASS versions for screening purposes. Provides an applied decision-making framework summarizing recommended DASS versions according to assessment goals, time constraints, and psychometric trade-offs.

**Supplementary Table S1. Conceptual mapping of DASS-21 items across abbreviated versions**

| DASS-21 Item No. | Item content (Spanish) | Theoretical dimension | DASS-21 | DASS-14 | DASS-12 | DASS-9 | DASS-8 |
| --- | --- | --- | --- | --- | --- | --- | --- |
| 1 | Encontré difícil calmarme/relajarme | Stress | ✓ | ✓ | — | — | — |
| 2 | Estuve consciente de la resequedad en mi boca | Anxiety | ✓ | — | — | — | — |
| 3 | Al parecer no experimenté algún sentimiento positivo | Depression | ✓ | ✓ | — | — | — |
| 4 | Tuve problemas para respirar (respiración muy rápida o falta de aire en ausencia de esfuerzo físico) | Anxiety | ✓ | ✓ | — | — | — |
| 5 | Encontré difícil tener la iniciativa para hacer las cosas | Depression | ✓ | — | ✓ | ✓ | — |
| 6 | Tendí a sobrereaccionar ante situaciones | Stress | ✓ | ✓ | ✓ | ✓ | — |
| 7 | Experimenté temblores (por ejemplo, en las manos) | Anxiety | ✓ | ✓ | ✓ | ✓ | — |
| 8 | Sentí que tuve nervios | Stress | ✓ | — | — | — | ✓ |
| 9 | Me preocupé por situaciones que me asustan y puedo quedar en ridículo | Anxiety | ✓ | — | ✓ | ✓ | ✓ |
| 10 | Sentí que no tenía nada que esperar | Depression | ✓ | ✓ | ✓ | ✓ | ✓ |
| 11 | Me di cuenta de que me ponía nerviosa/o | Stress | ✓ | ✓ | ✓ | ✓ | — |
| 12 | Encontré difícil relajarme | Stress | ✓ | ✓ | ✓ | — | ✓ |
| 13 | Me sentí desanimado y triste | Depression | ✓ | — | — | — | ✓ |
| 14 | Estuve intolerante con todo lo que me distrajera de lo que estaba haciendo | Stress | ✓ | ✓ | ✓ | ✓ | — |
| 15 | Sentí que estuve a punto de entrar en pánico | Anxiety | ✓ | — | ✓ | ✓ | ✓ |
| 16 | Fui incapaz de entusiasmarme por algo | Depression | ✓ | ✓ | ✓ | ✓ | ✓ |
| 17 | Sentí que no valía como persona | Depression | ✓ | ✓ | ✓ | — | — |
| 18 | Sentí que estaba muy irritable | Stress | ✓ | ✓ | — | — | — |
| 19 | Sentí los latidos de mi corazón a pesar de no haber hecho esfuerzo físico (ej., aumento o enlentecimiento del ritmo cardiaco) | Anxiety | ✓ | ✓ | — | — | — |
| 20 | Sentí miedo sin alguna razón | Anxiety | ✓ | — | ✓ | — | ✓ |
| 21 | Consideré que la vida no tenía sentido | Depression | ✓ | ✓ | — | — | — |

**Note.** Item–dimension assignment follows the original theoretical structure of the DASS. Checkmarks indicate item inclusion in previously published abbreviated versions. This table documents conceptual and structural correspondence across versions and does not reflect empirical item–factor loadings estimated in the present study. Item wording is shown in the Spanish version administered in this study. English translations are intentionally omitted to preserve semantic fidelity and avoid introducing interpretive variability.

**Supplementary Table S2. Item-level descriptive statistics (mean, SD, skewness, and kurtosis) for the DASS-21**

| Item | **Mean** | **SD** | **Skewness** | **Kurtosis** |
| --- | --- | --- | --- | --- |
| 1. Encontré difícil calmarme/relajarme | 1.1 | 0.8 | 0.5 | -0.2 |
| 2. Estuve consciente de la resequedad en mi boca | 1.1 | 1.0 | 0.5 | -0.9 |
| 3. Al parecer no experimenté algún sentimiento positivo | 0.6 | 0.8 | 1.2 | 0.8 |
| 4. Tuve problemas para respirar (respiración muy rápida o falta de aire en ausencia de esfuerzo físico) | 0.5 | 0.8 | 1.5 | 1.6 |
| 5. Encontré difícil tener la iniciativa para hacer las cosas | 1.0 | 0.9 | 0.6 | -0.3 |
| 6. Tendí a sobrereaccionar ante situaciones | 1.0 | 0.9 | 0.7 | -0.5 |
| 7. Experimenté temblores (por ejemplo, en las manos) | 0.7 | 0.9 | 1.2 | 0.3 |
| 8. Sentí que tuve nervios | 1.5 | 0.9 | 0.1 | -0.9 |
| 9. Me preocupé por situaciones que me asustan y puedo quedar en ridículo | 1.2 | 1.0 | 0.4 | -1.0 |
| 10. Sentí que no tenía nada que esperar | 0.7 | 0.9 | 1.2 | 0.5 |
| 11. Me di cuenta de que me ponía nerviosa/o | 1.5 | 1.0 | 0.2 | -0.9 |
| 12. Encontré difícil relajarme | 1.0 | 0.9 | 0.6 | -0.3 |
| 13. Me sentí desanimado y triste | 0.9 | 0.9 | 0.7 | -0.2 |
| 14. Estuve intolerante con todo lo que me distrajera de lo que estaba haciendo | 0.9 | 0.9 | 0.7 | -0.4 |
| 15. Sentí que estuve a punto de entrar en pánico | 0.5 | 0.9 | 1.5 | 1.3 |
| 16. Fui incapaz de entusiasmarme por algo | 0.6 | 0.9 | 1.4 | 1.0 |
| 17. Sentí que no valía como persona | 0.4 | 0.8 | 1.9 | 2.9 |
| 18. Sentí que estaba muy irritable | 1.0 | 1.0 | 0.7 | -0.7 |
| 19. Sentí los latidos de mi corazón a pesar de no haber hecho esfuerzo físico (Ejemplo: Sentir aumento del ritmo cardiaco o que va más lento) | 0.7 | 0.9 | 1.2 | 0.3 |
| 20. Sentí miedo sin alguna razón | 0.7 | 0.9 | 1.2 | 0.4 |
| 21. Consideré que la vida no tenia sentido | 0.3 | 0.7 | 2.6 | 6.2 |

**Note.** Positive skewness reflects the low endorsement of severe symptoms in this non-clinical university sample. Items assessing more severe depressive content (e.g., Items 17 and 21) show elevated kurtosis, a pattern commonly observed in general population samples and consistent with the use of ordinal estimators in subsequent factor analyses.

**Supplementary Table S3. Model fit indices for unidimensional, hierarchical, and bifactor models of the DASS (N = 1,251)**

| Version | Model | χ² | df | CFI | TLI | RMSEA [90% CI] | SRMR | ΔCFI | ΔRMSEA |
| --- | --- | --- | --- | --- | --- | --- | --- | --- | --- |
| DASS-21 | Unidimensional | — | — | .990 | .988 | .068 | .052 | — | — |
|  | Hierarchical | — | — | .990 | .988 | .068 [.064–.071] | .052 | .000 | .000 |
|  | **Bifactor** | — | — | **.997** | **.996** | **.022 [.017–.027]** | **.037** | **+.007** | **−.046** |
| DASS-14 | Unidimensional | — | — | .992 | .990 | .036 [.030–.042] | .046 | — | — |
|  | Hierarchical | — | — | .988 | .985 | .076 [.071–.082] | .052 | −.009 | +.054 |
|  | **Bifactor** | — | — | **.996** | **.994** | **.029 [.022–.036]** | **.037** | **+.008** | **−.047** |
| DASS-12 | Unidimensional | — | — | .996 | .995 | .025 [.017–.034] | .035 | — | — |
|  | Hierarchical | — | — | .996 | .995 | .046 [.039–.053] | .037 | .000 | +.021 |
|  | **Bifactor** | — | — | **.998** | **.997** | **.019 [.006–.029]** | **.028** | **+.002** | **−.006** |
| DASS-9 | Unidimensional | — | — | .993 | .990 | .037 [.027–.048] | .038 | — | — |
|  | Hierarchical | — | — | .993 | .989 | .061 [.052–.072] | .043 | .000 | +.024 |
|  | **Bifactor** | — | — | **.996** | **.992** | **.033 [.020–.046]** | **.031** | **+.003** | **−.004** |
| DASS-8 | Unidimensional | — | — | .997 | .995 | .030 [.017–.044] | .033 | — | — |
|  | Hierarchical | — | — | .997 | .994 | .057 [.045–.069] | .034 | .000 | +.027 |
|  | **Bifactor** | — | — | **.997** | **.994** | **.033 [.017–.049]** | **.028** | **.000** | **+.003** |

### **Note.** Models were estimated using the WLSMV estimator based on polychoric correlation matrices. ΔCFI and ΔRMSEA indicate changes relative to the unidimensional model within each DASS version. CFI = Comparative Fit Index; TLI = Tucker–Lewis Index; RMSEA = Root Mean Square Error of Approximation; SRMR = Standardized Root Mean Square Residual. Bolded values indicate the best-fitting solution for each DASS version.

**Supplementary Table S4. Item–total correlations and McDonald’s omega if item deleted across DASS**

|  | ω if item deleted (DASS-21) | | | | | Item–total correlation | | | | |
| --- | --- | --- | --- | --- | --- | --- | --- | --- | --- | --- |
| Item | **DASS 21** | **DASS 14** | **DASS 12** | **DASS 9** | **DASS 8** | **DASS**  **21** | **DASS 14** | **DASS 12** | **DASS 9** | **DASS 8** |
| 1. Encontré difícil calmarme/relajarme | 0.94 | 0.91 |  |  |  | 0.62 | 0.61 |  |  |  |
| 2. Estuve consciente de la resequedad en mi boca | 0.94 |  |  |  |  | 0.38 |  |  |  |  |
| 3. Al parecer no experimenté algún sentimiento positivo | 0.94 | 0.91 |  |  |  | 0.48 | 0.50 |  |  |  |
| 4. Tuve problemas para respirar (respiración muy rápida o falta de aire en ausencia de esfuerzo físico) | 0.94 | 0.91 |  |  |  | 0.60 | 0.59 |  |  |  |
| 5. Encontré difícil tener la iniciativa para hacer las cosas | 0.94 |  | 0.90 | 0.86 |  | 0.54 |  | 0.53 | 0.52 |  |
| 6. Tendí a sobrereaccionar ante situaciones | 0.94 | 0.91 | 0.90 | 0.86 |  | 0.60 | 0.59 | 0.58 | 0.57 |  |
| 7. Experimenté temblores (por ejemplo, en las manos) | 0.94 | 0.91 | 0.90 | 0.86 |  | 0.61 | 0.60 | 0.58 | 0.56 |  |
| 8. Sentí que tuve nervios | 0.94 |  |  |  | 0.87 | 0.67 |  |  |  | 0.66 |
| 9. Me preocupé por situaciones que me asustan y puedo quedar en ridículo | 0.93 |  | 0.90 | 0.85 | 0.87 | 0.66 |  | 0.67 | 0.66 | 0.67 |
| 10. Sentí que no tenía nada que esperar | 0.94 | 0.91 | 0.90 | 0.86 | 0.88 | 0.64 | 0.62 | 0.64 | 0.62 | 0.63 |
| 11. Me di cuenta de que me ponía nerviosa/o | 0.94 | 0.91 | 0.90 | 0.85 |  | 0.69 | 0.63 | 0.68 | 0.67 |  |
| 12. Encontré difícil relajarme | 0.93 | 0.90 | 0.90 |  | 0.87 | 0.74 | 0.72 | 0.71 |  | 0.70 |
| 13. Me sentí desanimado y triste | 0.94 |  |  |  | 0.87 | 0.70 |  |  |  | 0.67 |
| 14. Estuve intolerante con todo lo que me distrajera de lo que estaba haciendo | 0.94 | 0.91 | 0.90 | 0.85 |  | 0.64 | 0.64 | 0.62 | 0.61 |  |
| 15. Sentí que estuve a punto de entrar en pánico | 0.93 |  | 0.90 | 0.85 | 0.87 | 0.72 |  | 0.70 | 0.66 | 0.69 |
| 16. Fui incapaz de entusiasmarme por algo | 0.94 | 0.91 | 0.90 | 0.86 | 0.88 | 0.58 | 0.59 | 0.57 | 0.55 | 0.54 |
| 17. Sentí que no valía como persona | 0.94 | 0.91 | 0.90 |  |  | 0.64 | 0.65 | 0.63 |  |  |
| 18. Sentí que estaba muy irritable | 0.94 | 0.90 |  |  |  | 0.68 | 0.68 |  |  |  |
| 19. Sentí los latidos de mi corazón a pesar de no haber hecho esfuerzo físico (Ejemplo: Sentir aumento del ritmo cardiaco o que va más lento) | 0.94 | 0.90 |  |  |  | 0.69 | 0.67 |  |  |  |
| 20. Sentí miedo sin alguna razón | 0.94 |  | 0.90 |  | 0.87 | 0.70 |  | 0.68 |  | 0.68 |
| 21. Consideré que la vida no tenia sentido | 0.94 | 0.91 |  |  |  | 0.55 | 0.56 |  |  |  |

**Note.** Empty cells indicate that the item is not included in the corresponding abbreviated DASS version. McDonald’s omega if item deleted is reported for the total score of each version. Item–total correlations correspond to corrected item–total correlations.

**Supplementary Table S5a. Bifactor reliability and dimensionality indices across DASS versions**

| Version | ECV | ωH | PUC | ωHS Stress | ωHS Anxiety | ωHS Depression | H Stress | H Anxiety | H Depression |
| --- | --- | --- | --- | --- | --- | --- | --- | --- | --- |
| DASS-21 | 0.81 | 0.88 | 0.70 | 0.01 | 0.09 | 0.23 | 0.03 | 0.23 | 0.41 |
| DASS-14 | 0.77 | 0.84 | 0.69 | 0.03 | 0.14 | 0.25 | 0.08 | 0.25 | 0.38 |
| DASS-12 | 0.84 | 0.85 | 0.73 | 0.02 | 0.04 | 0.19 | 0.05 | 0.09 | 0.29 |
| DASS-9 | 0.83 | 0.82 | 0.75 | 0.02 | 0.02 | 0.18 | 0.04 | 0.04 | 0.25 |
| DASS-8 | 0.86 | 0.83 | 0.75 | 0.01 | 0.00 | 0.19 | 0.01 | 0.01 | 0.28 |

**Note. ECV = explained common variance attributable to the general factor; ωH = omega hierarchical for the general factor; PUC = proportion of uncontaminated correlations; ωHS = omega hierarchical subscale. Higher ECV and ωH values indicate greater dominance of the general distress factor. Low ωHS values reflect limited unique variance retained by specific factors after accounting for the general factor, a pattern that becomes more pronounced as the number of items decreases.**

**Supplementary Table S5b. Reliability indices for hierarchical DASS models**

| Version | ω Stress | ω Anxiety | ω Depression | H Stress | H Anxiety | H Depression |
| --- | --- | --- | --- | --- | --- | --- |
| DASS-21 | 0.62 | 0.56 | 0.59 | 0.92 | 0.90 | 0.91 |
| DASS-14 | 0.60 | 0.60 | 0.62 | 0.90 | 0.82 | 0.89 |
| DASS-12 | 0.55 | 0.62 | 0.59 | 0.83 | 0.87 | 0.85 |
| DASS-9 | 0.51 | 0.59 | 0.54 | 0.76 | 0.81 | 0.78 |
| DASS-8 | 0.62 | 0.66 | 0.61 | 0.77 | 0.85 | 0.83 |

**Note. ω coefficients reflect the reliability of first-order subscales within hierarchical models. H coefficients indicate construct replicability. Although reliability of specific subscales decreases as item counts are reduced, H values suggest that the underlying constructs remain moderately to well defined across versions.**

**Supplementary Table S6. Spearman correlations between the DASS-21 and its abbreviated versions (N = 1,251)**

| Dimension | DASS-14 | DASS-12 | DASS-9 | DASS-8 |
| --- | --- | --- | --- | --- |
| Estrés | .988*** | .966*** | .927*** | .892*** |
| Ansiedad | .905*** | .919*** | .895*** | .871*** |
| Depresión | .928*** | .941*** | .920*** | .918*** |
| Total | .977* | .976* | .956* | .950* |

**Note.** Values are Spearman’s rank-order correlations (ρ) between DASS-21 subscale and total scores and the corresponding scores from abbreviated versions (14, 12, 9, and 8 items). All correlations were statistically significant at *p* < .001. Correlations ≥ .95 were interpreted as evidence of functional equivalence between versions, following established criteria. Given partial item overlap across forms, these associations indicate preservation of essential construct-related information rather than independent validity.

## **Supplementary Table S7. Practical guide for selecting DASS versions for screening purposes**

| Applied need | Recommended version | Psychometric rationale | Notes / cautions |
| --- | --- | --- | --- |
| Rapid screening of global emotional distress under severe time constraints | DASS-9 / DASS-8 | Strong dominance of the general distress factor; adequate bifactor fit; high measurement efficiency | Differentiation between stress, anxiety, and depression is limited |
| Institutional monitoring of student well-being | DASS-12 | Balanced trade-off between parsimony, dimensional differentiation, and external validity | Dimensional scores should be interpreted with caution |
| Assessment focused on differential profiles (stress, anxiety, depression) | DASS-12 / DASS-21 | Retain greater specific factor variance compared to ultra-brief versions | Higher respondent burden than ultra-short forms |
| Group comparisons (e.g., sex) | DASS-12 | Evidence of scalar measurement invariance across sex | Allows valid comparisons of latent means |
| Use within extensive test batteries including multiple constructs | DASS-12 | Substantial item reduction with no meaningful loss of psychometric information | Preferable to DASS-21 in applied and time-limited settings |
| Detailed structural or psychometric research | DASS-21 | Broader content coverage and greater dimensional stability | Lower efficiency for large-scale screening applications |

**Note**. Recommendations are derived from the structural, reliability, and equivalence evidence reported in the present study (Supplementary Tables S3–S5). This guide is intended for screening and applied decision-making purposes and does not imply strict score equivalence or full interchangeability across DASS versions. Selection should be guided by the specific assessment purpose, time constraints, and required level of dimensional resolution.
